# Autophagic Marker *MAP1LC3B* Expression Levels Are Associated with Carotid Atherosclerosis Symptomatology

**DOI:** 10.1371/journal.pone.0115176

**Published:** 2014-12-12

**Authors:** Bhairavi Swaminathan, Haize Goikuria, Reyes Vega, Alfredo Rodríguez-Antigüedad, Antonio López Medina, María del Mar Freijo, Koen Vandenbroeck, Iraide Alloza

**Affiliations:** 1 Neurogenomiks, Neurosciences Department, Faculty of Medicine and Odontology, University of Basque Country, Leioa, Spain; 2 IKERBASQUE, Basque Foundation for Sciences, Bilbao, Spain; 3 Achucarro Basque Center for Neurosciences, Zamudio, Spain; 4 Department of Neurology, Basurto Hospital, Bilbao, Spain; Heart Research Institute, Australia

## Abstract

**Objectives:**

The mechanism by which atheroma plaque becomes unstable is not completely understood to date but analysis of differentially expressed genes in stable versus unstable plaques may provide clues. This will be crucial toward disclosing the mechanistic basis of plaque instability, and may help to identify prognostic biomarkers for ischaemic events. The objective of our study was to identify differences in expression levels of 59 selected genes between symptomatic patients (unstable plaques) and asymptomatic patients (stable plaques).

**Methods:**

80 carotid plaques obtained by carotid endarterectomy and classified as symptomatic (>70% stenosis) or asymptomatic (>80% stenosis) were used in this study. The expression levels of 59 genes were quantified by qPCR on RNA extracted from the carotid plaques obtained by endarterectomy and analyzed by means of various bioinformatic tools.

**Results:**

Several genes associated with autophagy pathways displayed differential expression levels between asymptomatic and symptomatic (i.e. *MAP1LC3B*, *RAB24*, *EVA1A*). In particular, mRNA levels of *MAP1LC3B*, an autophagic marker, showed a 5−fold decrease in symptomatic samples, which was confirmed in protein blots. Immune system−related factors and endoplasmic reticulum-associated markers (i.e. *ERP27*, *ITPR1*, *ERO1LB, TIMP1, IL12B*) emerged as differently expressed genes between asymptomatic and symptomatic patients.

**Conclusions:**

Carotid atherosclerotic plaques in which *MAP1LC3B* is underexpressed would not be able to benefit from *MAP1LC3B*−associated autophagy. This may lead to accumulation of dead cells at lesion site with subsequent plaque destabilization leading to cerebrovascular events. Identified biomarkers and network interactions may represent novel targets for development of treatments against plaque destabilization and thus for the prevention of cerebrovascular events.

## Introduction

Atherosclerosis in the carotid artery is the second leading cause of death and the third cause of disability-adjusted life-years worldwide [1], [2]. Carotid atherosclerosis is a disorder with an important inflammatory component and is considered a risk factor for developing a cerebrovascular accident. A high stenosis grade is a risk factor for a cerebrovascular event but, since it is known that a percentage of patients with high stenosis will present asymptomatic plaques [3], stenosis alone is not sufficient for identification of patients at risk. In contrast, plaques from symptomatic patients are more likely to become unstable and predisposed to rupture [4]. The rupture and destabilization of the plaque in the carotid artery can lead to an ischemic attack [5]. However, the precise mechanisms by which atheroma plaque becomes unstable [6] are still unknown.

Several clinical and pathological studies have revealed specific gene expression biomarkers associated with plaque rupture among symptomatic patients. For instance, matrix metalloproteinase−1 (MMP1) and MMP12, and CD163 and HO−1 have been identified as potential indicators of carotid plaque instability [7], [8]. In addition, ADAMDEC1, MMP9 and legumain genes have been described as over−expressed genes in unstable areas of carotid plaques when compared with stable areas of the same plaque [9]. More recently, IL17A has also been associated with vulnerability of the atheroma plaque [10], while a microarray-based study comparing gene expression levels between symptomatic and asymptomatic patients identified ten genes with significant differences between the two groups [11]. Thus, even if various genes have been suggested to play a role in plaque destabilization, further studies are needed to gain a more comprehensive understanding of the process.

The aim of this study was to perform an extended candidate gene expression analysis in a collection of 80 atheroma sample collection both to identify novel biomarkers and to validate previously reported associated markers. We analyzed 59 genes including 9 genes reported before to be involved in atherogenesis [8], [9], [12], [13], [14], [15], [16], 10 cytokine genes [17], [18], [19], in addition to 40 genes related with endoplasmic reticulum pathways and cellular stress [20], [21]. Our study provides further insight into the mechanism of plaque destabilization associated with cerebrovascular events.

## Materials and Methods

### Patients and endarterectomy

Patients were recruited from the department of Neurology, Basurto Hospital (Bilbao, Spain) to undergo carotid endarterectomy (CEA). CEA was performed in patients who presented a degree of stenosis higher than 70% with previous history of transient ischemic attack or ipsilateral stroke (symptomatic) or higher than 80% without any presence of cerebrovascular events (asymptomatic). Quantification of degree (%) of stenosis was performed with carotid cervical Eco-Doppler ultrasound and angioresonance imaging vs angio CT according to established criteria [1]. Demographic and clinical data for these patients are summarized in Table 1. This study was approved by the local ethical committee (Ethical Committee of Clinical Research, Basurto Hospital) and all carotid atheroma plaques were collected from patients who had signed written informed consent. This research was performed in agreement with the principles outlined in the Declaration of Helsinki. The CEA plaques were paraffin-embedded and frozen at -80°C until further use.

**Table 1:** Demographic characteristics of patients included in the study.

Patient characteristics	All	Symptomatic	Asymptomatic
Number, n	80	45	35
Age, years ± SD	68±8	68±8	67±8
Sex M/F, n	70/9	39/5	31/4
***Risks Factors (%)***			
Contralateral occlusion	19	18	20
Hipertensive	59.5	56.8	63
DM (diabetes mellitus)	27.8	27	28,5
Cholesterol	45.5	41	51
Cardiopaty	28	23	34
Isquemic cardiopaty	19	13.6	26
ATF (atrial fibrillation)	5	4.5	5.7
Intermittent claudication	34	25	46
Tobacco	36.7	34	40
***Medications (%)***			
Statin	21.5	18.8	26
Anticoagulant	6.3	6.8	6

### RNA extraction and reverse transcription

Frozen carotid atheroma plaque samples were immersed in Ambion RNA*later*-ICE (Life technologies, UK) and placed overnight at −20°C. Plaques were homogenized following the manufacture's instructions of TRIzol (Life technologies) and the RNA was extracted with the Ambion RiboPure Kit (Life technologies). The purity of RNA samples was estimated with the Nanodrop (Thermo scientific) using the ratio of absorbance values at 260 nm and 280 nm. 250 µg of extracted RNA were retrotranscribed with the High Capacity cDNA Reverse Transcription Kits from ABI (Life technologies) on the Veriti fast thermal cycler (Life technologies) following the manual instructions. The integrity of RNA (18S and 28S rRNA) was verified by 1% agarose gel electrophoresis.

### Selection of genes

Genes selected for this study are candidates for involvement in the carotid atherosclerotic processes associated with symptomatology. Literature was scrutinized to identify potential novel pathways involved in the instability of the plaque on the basis of which a total of 59 candidate genes were selected. Nine of these genes were known to be involved in atherogenesis (*VCAM1*, *CD163*, *MMP9*, *TIMP1*, *COL3A1*, *THBS1*, *EDN1*, *ELANE*, *ELN*) [8], [9], [12], [13], [14], [15], [16], another 10 genes were related to the immune system (*IL12B*, *IL12A*, *TNF*, *IL10*, *IL17A*, *IL18*, *IL23A*, *IL6*, *TGFB1* and *IL1A*) [17], [18], [19] and the remaining 40 were selected for their involvement in endoplasmic reticulum (ER)−related pathways or cellular stress (i.e. *CALR, DDIT3, ERO1LB*, etc.) [20], [21] (S1 Table).

### Real-time qPCR

SYBR green technology was used to perform Real Time qPCR. Validated specific primers for genes of interest and house keeping genes (β−actin and *GAPDH*) were purchased from Qiagen (QuantiTect Primer assays) (S1 Table). For each sample we performed SYBR green real-time qPCR in quadruplicates using the *Power*SYBR Green Master Mix on the ABI7500fast detection system (Life Technologies) according to manufacture's instructions. The amplification protocol included a melting curve dissociation step to confirm the inexistence of nonspecific amplification products. The normalization of the gene expression data was performed using the geometric mean of the two house-keeping genes (β−actin and *GAPDH*). The geometric mean of 2 or more selected housekeeping genes has been validated as a normalization method for qPCR data [22]. The analysis was performed using the comparative Ct method (2^−ΔΔCt^) and the fold change was calculated from normalized Ct values. The statistical significance of fold change differences between the symptomatic and asymptomatic groups was calculated with the non-parametric Mann-Whitney U test and the level of significance was set at P<0.05. PCR amplification efficiency was found close to 100% in all cases.

### Bioinformatics enrichment and correlation analysis

Enrichment clustering analysis was performed using the GeneCodis 3.0 program (http://genecodis.cnb.csic.es/), which enables identification of combinations of significant annotations associated with the analyzed gene list. A statistical discrete probability distribution function test (hypergeometric distribution) was used in the enrichment clustering analysis and the P values were adjusted for multiple tests using the false discovery rate method of Benjamini and Hochberg with the cut-off threshold for significance set at 0.001. Spearmann's correlation test was performed using GrapPad version 5.0 (GraphPad Software, La Jolla, CA) to facilitate the identification of interrelated markers and P<0.05 was considered significant.

### Protein isolation and western blot

0.02 g of carotid atheroma plaque was washed with PBS and cut at 300 µm with McIllwain Tissue Chopper (The Mickle Laboratory Engineering Co. LTD.) and the resulting mixture was diluted in 100 µl RIPA buffer (150 mM NaCl; 50 mM Tris-Cl, pH 7.5; 1% NP-40; 0.5% deoxycholate; 0.1% sodium dodecyl sulphate) containing protease inhibitors. Samples were homogenized for 1 h and 30 min on a rotator at 4°C followed by centrifugation for 15 min at 14800 rpm. The supernatants were collected and 10 µl of sample was subjected to 15% SDS-PAGE. Proteins were electrophoretically transferred to a PVDF membrane and blocked overnight. Then, membranes were incubated with rabbit anti-LC3B (D11) antibody (Cell Signalling) or mouse anti-GAPDH (6C5) (Millipore) followed by incubation with anti-rabbit or anti-mouse horseradish peroxidase (HRP) conjugate secondary antibody. Bound antibodies were detected with SuperSignal substrate (Thermo Scientific) on a Chemidoc detection system (BioRad). Signals were quantified by densitometric scanning with the Chemidoc software and densitometric values were normalized against GAPDH. Statistical significance was determined by using the non parametric Mann-Whitney U test.

## Results

### Gene expression profile of symptomatology within carotid plaques

A total of 35 asymptomatic and 45 symptomatic plaques obtained after CEA were tested for differential expression using the comparative Ct method. The demographic and clinical characteristics of the studied group are shown on Table 1.

Quantitative RT-PCR test data analysis based on the comparative Ct method revealed differential expression levels higher than 1.4 fold change (FC) for 25 of the 59 genes scrutinized upon comparison of symptomatic versus asymptomatic (S vs A) and asymptomatic versus symptomatic (A vs S) (Table 2 and Table 3). From the 25 identified differentially expressed genes (FC≥1.4), 15 showed a significant FC (P<0.05) (i.e. *TIMP1*, *ITPR1*, *CD163*, *ERP29*, *EVA1A*, *PARK2*, *MMP9*, *HSP1A1*, *SEC63*, *ERO1LB*, *RAB24*, *LMAN1*, *IL12B*, *ERP27*, *MAP1LC3B*). From this list, *MAP1LC3B* was uncovered as the gene showing the highest fold difference between the asymptomatic and symptomatic plaques (FC = 5) with a significance level of P<0.0001. In this study we included also genes that have been reported previously to be differentially expressed in carotid plaques upon comparison of symptomatic versus asymptomatic samples (S vs A). This confirmed that *CD163* is upregulated in symptomatic plaques (FC = 1.81, P = 0.044) [8]. In addition, we confirmed *HMOX1* and *MMP9* in our group of samples to be overexpressed (S vs A) with trends towards significance (FC = 2.07, P = 0.065 and FC = 1.4, P = 0.0505 respectively) [8], [9].

**Table 2:** Ranking of gene expression markers according to the highest fold change in symptomatic (S) compared with asymptomatic (A) samples quantified by real time RT−PCR.

Gene name	FC (S vs A)	P-value
TIMP metallopeptidase inhibitor 1 (*TIMP1*)	**3.45**	0.032*
Inositol 1,4,5-triphosphate repector, type 1 (*ITPR1*)	**2.83**	0.037*
Heme oxygenase (decycling) 1 (*HMOX1*)	**2.07**	0.065
CD163 molecule/Hemoglobin scavenger receptor (*CD163)*	**1.81**	0.044*
Mitogen-activated protein kinase 1 (*MAPK1*)	**1.75**	0.057
Endoplasmic reticulum protein 29 (*ERP29*)	**1.68**	0.031*
Mesencephalic astrocyte-derived neurotrophic factor (*MANF*)	**1.68**	0.48
Eva-1 homolog A (C. Elegans) (*EVA1A*)	**1.57**	0.033*
Transforming growth factor, beta 1 (*TGFB1*)	**1.57**	0.13
Parkinson protein 2, E3 ubiquitin protein ligase (*PARK2*)	**1.52**	0.043*
Protein disulfide isomerase family A, member 6 (*PDIA6*)	**1.47**	0.093
Collagen, type III, alpha 1 (*COL3A1*)	**1.42**	0.063
Matrix metallopeptidase 9 (gelatinase B) (*MMP9*)	**1.4**	0.05*

The statistical significance was analyzed with the non-parametrical statistical test Mann-Whitney U test (* P≤0.05 and ** P≤0.0001).

**Table 3:** Ranking of gene expression markers according to highest fold change in asymptomatic (A) compared with symptomatic (S) samples quantified by Real Time RT−PCR.

Gene name	FC (A vs S)	P-value
Microtubule-associated protein 1 light chain 3 beta (*MAP1LC3B*)	**5**	<0.0001**
Endoplasmic reticulum protein 27 (*ERP27*)	**4.07**	0.047*
Interleukin 12B (*IL12B*)	**2.5**	0.028*
Lectin, mannose-binding, 1 (*LMAN1*)	**2**	0.04*
RAB24, member RAS oncogene family (*RAB24*)	**1.75**	0.031*
ERO1-like beta (S.cerevisiae) (*ERO1LB*)	**1.67**	0.034*
v-raf-1 murine leukemia viral oncogene homolog 1 (*RAF1*)	**1.67**	0.074
Protein disulfide isomerase family A, member 4 (*PDIA4*)	**1.52**	0.062
SEC63 homolog (S. cerevisiae) (*SEC63*)	**1.46**	0.05*
Heat shock 70 kDa protein 1A (*HSPA1A*)	**1.46**	0.024*
Stress-associated endoplasmic reticulum protein 1 (*SERP1*)	**1.4**	0.32
DnaJ (Hsp40) homolog, subfamily B, member 9 (*DNAJB9*)	**1.4**	0.067

The statistical significance was analyzed with the non-parametrical statistical test Mann-Whitney U test (* P≤0.05 and ** P≤0.0001).

In order to identify functional relationships among the differentially expressed genes between the symptomatic and asymptomatic patients, we applied the software GeneCodis 3.0 for modular enrichment analysis that facilitated extraction of regulatory patterns with potential functional/biological significance. Twenty-four annotation groups (with at least 3 genes in each) obtained by including in the analysis the categories of Gene Ontology (biological process, cellular component and molecular function) and KEGG pathways (Kyoto Encyclopedia of Genes and Genomes) are shown in Table 4. Only the statistically corrected significant annotations are shown, with the corrected P−values obtained by hypergeometric analysis corrected by false discovery rate method. Every annotation group and the implicated genes are described with reference to their involved GO categories or pathways. The molecular enrichment analysis based specifically on KEGG pathways and GO molecular function revealed 9 groups of genes (formed of at least 3 genes) with significant concurrent annotations associated with 16 differentially expressed genes (Table 4). The significant concurrent annotations indicated pathways such as protein binding/protein folding chaperone (GO: 0005515), protein processing in the ER (KEGG: 04141), unfolded ER protein binding (GO: 0051082), infectious diseases (e.g. KEGG: 05140, 05145, 05142 and 05152), metal binding (GO: 0046872), pathways related with cancer (e.g. KEGG: 052220, 05200), or vascular smooth muscle contraction (KEGG: 04270).

**Table 4:** Molecular enrichment associations – GO: Molecular Functions and KEGG pathways.

Genes groups	Corrected P value	Concurrent Annotations
PARK2 (FC +1.52)	3.15×10^−4^	Metal ion binding (GO: 0046872)
LMAN1 (FC −2)		Protein binding (GO: 0005515)
RAF1 (FC −1.67)		
TIMP1 (FC +3.45)		
MMP9 (FC +1.4)		
MAPK1 (FC +1.75)	9.95×10^−16^	Protein binding (GO: 0005515)
RAF1 (FC −1.67)		Pathways in cancer (Kegg: 05200)
TGFB1 (FC +1.57)		
MMP9 (FC +1.4)		
MAPK1 (FC +1.75)	0.001	Protein binding (GO: 0005515)
RAF1 (FC −1.67)		MAPK signalling pathway (Kegg: 04010)
TGFB1 (FC +1.57)		Pathways in cancer (Kegg: 05200)
		Tuberculosis (Kegg: 05152)
		Renal cell carcinoma (Kegg: 05211)
		Chronic myeloid leukemia (Kegg: 05220)
		Colorectal cancer (Kegg: 05210)
		Pancreatic cancer (Kegg: 05212)
MAPK1 (FC +1.75)	0.001	Protein binding (GO: 0005515)
ITPR1 (FC +2.83)		Gap junction (Kegg: 04540)
RAF1 (FC −1.67)		Long-term depression (Kegg: 04730)
		Vascular smooth muscle contraction (Kegg: 04270)
		GnHR signalling pathway (Kegg: 04912)
		Long-term potentiation (Kegg: 04720)
MAPK1 (FC +1.75)	0.001	Protein binding (GO: 0005515)
RAF1 (FC −1.67)		Pathways in cancer (Kegg: 05200)
MMP9 (FC +1.4)		Bladder cancer (Kegg: 05219)
LMAN1 (FC −2)	0.001	Protein binding (GO: 0005515)
DNAJB9 (FC −1.38)		Unfolded protein binding (GO: 0051082)
HSPA1A (FC −1.46)		
LMAN1 (FC −2)	0.001	Protein processing in ER (Kegg: 04141)
SEC63 (FC −1.46)		Unfolded protein binding (GO: 0051082)
ERO1LB (FC −1.67)		
ERP29 (FC +1.68)	0.001	Protein processing in ER (Kegg: 04141)
PDIA6 (FC +1.47)		Protein disulfide isomerase activity (GO: 0003756)
PDIA4 (FC −1.52)		
MAPK1 (FC +1.75)	0.001	Leishmaniasis (Kegg: 05140)
IL12B (FC −2.5)		Toxoplasmosis (Kegg: 05145)
TGFB1 (FC +1.57)		Chagas disease (American trypanosomiasis) (Kegg: 05142)
		Tuberculosis (Kegg: 05152)

### Confirmation of gene expression pattern in an additional set of samples

In the course of the study, an additional set of 32 atheroma samples (10 asymptomatic and 22 symptomatic) were obtained by CEA from Basurto Hospital and we followed the procedure as before. Clinical data relative to this set of patients was similar to the patients who were included in the first analysis. We validated in this set a selection of genes, that had shown a significant (P<0.05) fold difference between the symptomatic and asymptomatic plaques and have biological functions of putative relevance to the plaque instability process. The following selected genes were tested in this cohort: *TIMP1*, *ITPR1*, *EVA1A1*, *COL3A1*, *ERO1LB*, *RAB24*, *LMAN1* and *MAP1LC3B*. The gene expression levels were analyzed by qPCR with SYBR green technology and we used the Mann-Whitney U test to calculate the P values. Results combining the original and validation sets of samples are shown in Table 5. The FC and P values for the genes tested were maintained exception made for *LMAN1*, whose significance was lost (Table 5). *MAP1LC3B* was confirmed as the gene showing the lowest FC of 6.13.

**Table 5:** Validation of selected markers in an extended sample set.^a^

Gene Symbol	FC (n = 112)	P-value
*TIMP1* (S vs A)	**5.1**	0.03*
*ITPR1* (S vs A)	**2.2**	0.0357*
*EVA1A* (S vs A)	**2.03**	0.041*
*COL3A1* (S vs A)	**1.65**	0.0039**
*MAP1LC3B* (A vs S)	**6.13**	<0.0001****
*EROL1B* (A vs S)	**2.15**	0.028*
*RAB24* (A vs S)	**1.84**	0.0026**
*LMAN1* (A vs S)	**1.3**	**n.s. (0.12)**

The statistical significance was analyzed with the non-parametrical statistical test Mann-Whitney U test (* P≤0.05 and **** P≤0.0001). FC; +, overexpressed and –, underexpressed).

a qPCR data analysis was performed in the combined set of the first (80 samples) and second cohorts (32 samples).

### MAP1LC3B protein expression analysis in carotid atherosclerotic plaques

**Figure 1:**
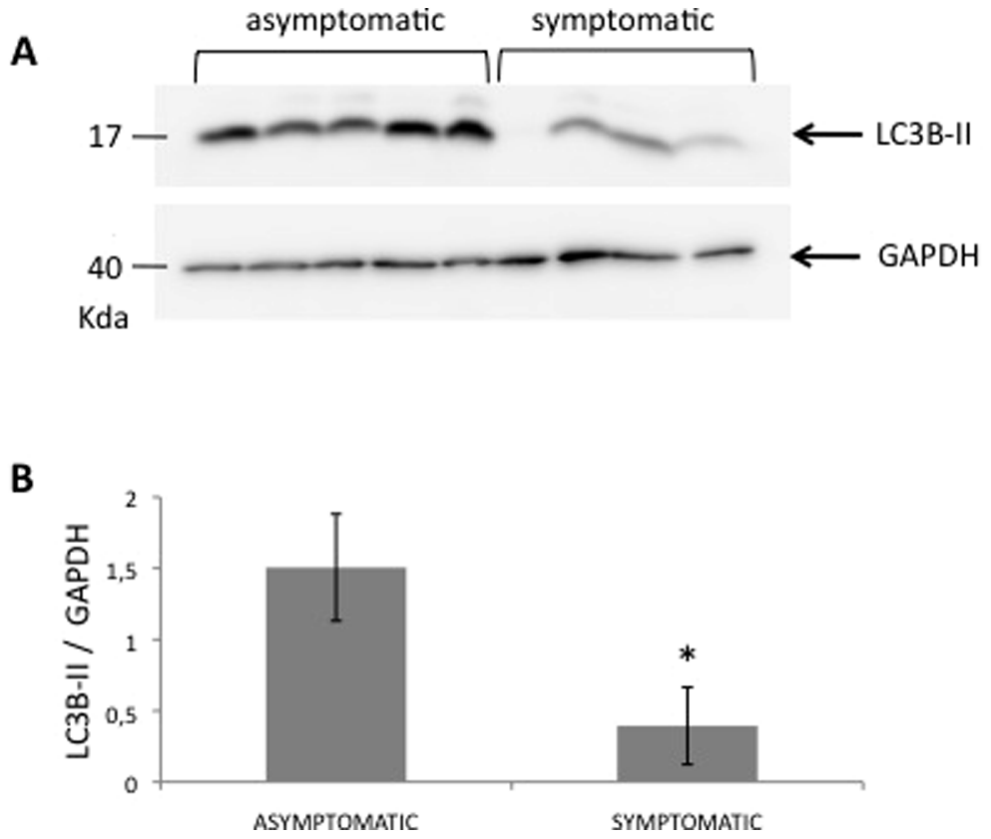
Differences in MAP1LC3B protein expression between symptomatic and asymptomatic. (A) MAP1LC3B and GAPDH carotid atheroma plaque protein levels were analyzed by Western blot and signal was detected on a ChemiDoc (XRS) detection system (BioRad). The blot shows the results from 5 asymptomatic and 4 symptomatic samples. (B) Densitometric analysis of western blot of LC3-II relative to GAPDH. *P = 0.015 symptomatic vs asymptomatic (Mann-Whitney U test).

Protein was extracted from 5 and 4 plaques from asymptomatic and symptomatic patients, respectively, and analyzed for MAP1LC3B levels by western blot. The MAP1LC3B antibody used reacts stronger with the band called LC3B II, which is indicative of autophagosome formation. Levels of LC3B II were significantly lower in symptomatic versus asymptomatic (Fig. 1A, B) suggesting that MAP1LC3B may play a functional role in preventing plaque destabilization.

### Correlation analysis: asymptomatic gene expression versus symptomatic gene expression

**Figure 2:**
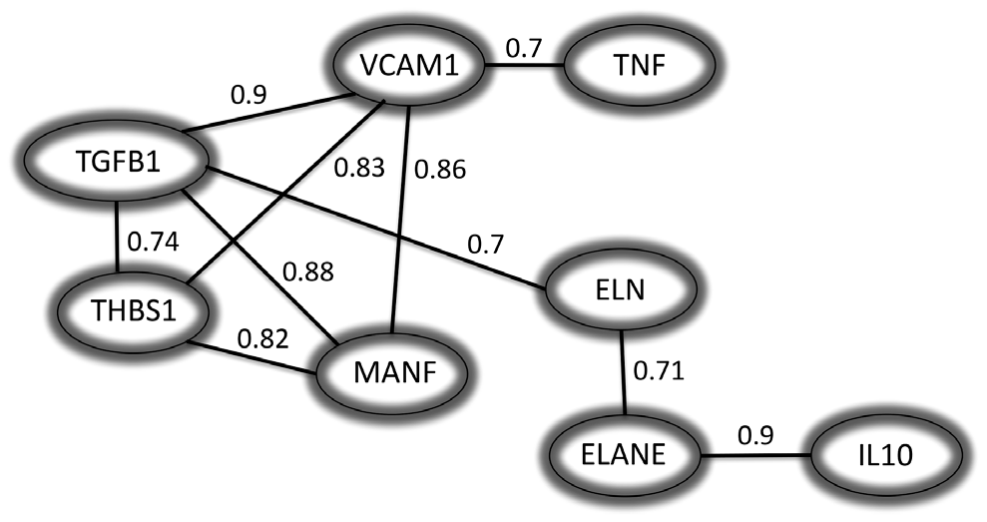
Correlation networks. The correlation has been computed as the normalised conditional mutual information. Only correlations above 0.7 are shown in this figure and with a significance of P<0.001.

Correlation analysis between each of the 59 of genes tested in this study was performed using Spearman's rank correlation test using the GraphPad Prism software version 5.0. *VCAM1* was correlated with *TGFB1* (r = 0.9), *MANF* (r = 0.86), *THBS1* (r = 0.83) and *TNF* (r = 0.7). On the other hand, *ELANE* was found to be correlated with *IL10* and *ELN* (r value of 0.9 and 0.71 respectively) while *ELN* was as well correlated with *TGFB1* (r = 0.7). Thus, the correlation analysis identified a pattern of genes that cluster together significantly. Fig. 2 shows correlation values between genes with significant (P<0.001) *r* value higher than 0.7.

## Discussion

The accumulation of atheroma plaque in the carotid artery can lead to stroke. The mechanisms by which a patient with an atherosclerotic plaque in the carotid artery develops ischemic stroke are not completely understood. However, the composition and the vulnerability of the atheroma plaque are important factors in the development of stroke [23], [24], [25]. In this study we adopted a gene expression analysis on carotid atheroma plaques extracted from symptomatic and asymptomatic patients of a series of genes, selected on the bases of literature search, so as to identify genes and/or cellular pathways that would aid in differentiating between the two groups studied and in understanding the mechanism/s that may be involved in this process. We were able to validate the gene expression patterns of previously reported genes (i.e. *MMP9*, *CD163* and *HMOX1*) [7], [8]. Importantly, we identified a group of previously unreported genes, which appeared differently expressed between the symptomatic and asymptomatic groups (*TIMP1*, *ITPR1*, *ERP27* or *MAP1LC3B*). These novel genes are primarily related with inflammation, autophagy, and ER related pathways.

*MAP1LC3B* emerged as the gene showing the most significant difference in FC between the two groups, with higher expression among asymptomatic patients. This gene has not been identified in previous human carotid plaque studies related with symptomatology. MAP1LC3B is involved in the recruitment of lipid droplets (cholesterol), which may promote autophagy [26]. MAP1LC3B−associated autophagy may be needed to clean up dead cells at the site of atherosclerotic lesions suggesting that autophagy induction could be beneficial in atherosclerosis [27], [28]. In addition, macrophage autophagy has been shown to play a protective role in advanced atherosclerosis [29]. Under hypoxic conditions, known to occur at the lesion site, the UPR (unfolded protein response) is activated as a protective mechanism by regulating the expression of MAP1LC3B [30], [31]. The high level of expression of *MAP1LC3B* in asymptomatic human carotid atherosclerotic plaques suggests a possible role for preventing the destabilization of the atherosclerotic plaque, probably by promoting basal autophagy activity at the lesion site [28], [32]. Besides, a proteomics study has identified MAP1LC3B as a protein indirectly related with plaque instability [33]. In addition, our data indicates that the nuclear protein high mobility group box 1 (*HMGB1*; FC = 1.3 (A vs S), P = 0.02), another factor involved in authophagy, may play a role in stimulating beneficial autophagy at the site of lesion. Although HMGB1 has been suggested to be involved in the progression of atherosclerotic plaque, both harmful and beneficial effects of HMGB1 have been documented [34], [35]. In particular, it has been described that HMGB1 regulates autophagy promoting programmed cell survival [36]. In addition, in our cohort we identified *RAB24* (FC = 1.75 (A vs S), P = 0.031), a protein considered to play a role in autophagy that colocalizes with MAP1LC3 in autophagosomes [37], to be underexpressed in symptomatic samples. On the other hand, eva-1 homolog A (C. Elegans) (*EVA1A;* FC = 1.57 (S vs A), P = 0.033) regulates apoptosis and autophagic cell death [38] and it has been described that high levels of EVA1A in rats with middle artery occlusion induced cellular damage conducting to cell death by lysosomal activation [39]. Therefore, *EVA1A* may play a role in symptomatic plaques by promoting plaque instability caused by autophagic cell death. Calcium homeostasis is also known to play a role in the cellular damage produced by ischemia [40]. Inositol 1,4,5-trisphosphate receptor type 1 (*ITPR1*; FC = 2.83 (S vs A), P = 0.037) is a channel involved in the influx of calcium from the ER into the cytosol [41]. Calcium release from the ER into the cytosol in basal conditions inhibits autophagy via AMP-activated protein kinase (AMPK) while during stress conditions the calcium signaling stimulates autophagy and apoptosis leading to cellular death [42]. Our results are in concordance with the hypothesis that induction of autophagy may be beneficial for plaque stabilization [43]. While autophagy is needed initially as a repair mechanism at the site of lesion in carotid atherosclerosis to eliminate damaged intracellular material, later on persisting cellular stress induces a type of cell death stimulated by autophagy. For that reason, targeting the later type of autophagy to prevent apoptosis/cell death would be the aim for avoiding the plaque disruption causing fatal symptoms to patients with carotid atherosclerosis.

ER stress-induced apoptosis is known be involved in vascular calcification [20] with its subsequent instability leading to cerebrovascular events [44]. Several differentially expressed genes identified in this study are associated with ER stress pathways (i.e. *ERP29, PDIA4*, *PDIA6*, *MAP1LC3B*, etc), mainly but not only associated with oxidative folding. In our recent study, ER stress induced by a non-coxib celecoxib analogue resulted in increased levels of *MAP1LC3B* [45], suggesting that the gene is regulated by unfolded protein response (UPR) pathways. The functional enrichment analysis performed, pointed as well to the ER as being associated with symptomatology. Protein binding/protein folding chaperone, protein processing in the ER, metal binding, cancer related pathways, infectious diseases and vascular smooth muscle contraction were biological functions that appeared to be significant in carotid atherosclerosis when we analyzed the 25 identified genes as differently expressed between the two groups.

In the early stage of the cellular stress development, Heat Shock 70 kD Protein−1A (HSPA1A) expression has been shown to exert protective effects by defending against apoptosis and by exerting an anti-inflammatory role [46]. Low levels of expression of *HSPA1A* (FC = 1.46, P = 0.024), as we observed in our symptomatic cohort, could indicate the initiation of inflammatory stage and cell death. Inflammation is accepted as one of the contributors of atherosclerosis with both the innate and acquired branches of the immune system playing a role in the process [47], [48]. However, our study is indicative for a protective effect displayed by several inflammation biomarkers associated with symptomatology of carotid disease. We identified several factors that seem to point to a beneficial effect of inflammation in asymptomatic patients. In particular, the cytokine subunits belonging to the IL12/IL23 family, *IL12B/p40* (FC = 2.5 (A vs S), P = 0.028) and *IL23A/p19* (FC = 1.18 (A vs S), P = 0.09), showed higher levels of expression in asymptomatic plaques. IL12B/IL23A forms the heterodimeric IL−23 cytokine that act as an inducer of the Th17 response. The Th17 response may be anti-atherogenic giving protection to patients in whom this response is induced by IL−23 [49], [17]. However, whilst the role of Th17 response in atherosclerosis has not yet been clarified entirely due to contradictory findings [18], [19], some authors have described its protective role in atherosclerosis [49], [50]. Similarly, our results would suggest a role for IL−23−induced Th17 response in carotid plaque stabilization.

In addition, in order to complement the gene expression analysis we attempted to correlate the expression of a gene to the expression of the other gene/s analysed in the carotid plaque samples. The interaction between genes in a network may indicate physical interaction or indirect regulation and it may possible to identify a subgroup of genes that regulate/interact with each other. This information could provide knowledge to develop new concepts for how the instability of plaque occurs. Here we identified groups of genes correlated with differently expressed genes (i.e. *VCAM1*, *TGFB1*, *THSB1*, *MANF*, *ELN, ELANE*, *IL10*, *TNF*). In this group of genes we observed correlation between the cytokine *IL10* and *ELANE*, an elastin protease known to degrade elastic fibers as elastin; indicating that elastin degradation and immune response process are common interacting regulatory mechanisms in atherosclerosis. Similarly, *VCAM1* correlation to *TNF* and *TGFB1* pointed out to an inflammatory pathway for these genes.

In conclusion, this study has identified several biomarkers with altered expression between symptomatic and as symptomatic samples, which are involved in inflammation, ER-related pathways and autophagy. Although the gene expression analysis performed with the technology used here or with similar technologies such as microarrays, have identified several markers associated with symptomatology, these technologies are limited to pre-selected genes. The application to unstable carotid atherosclerosis of the new emerging RNAseq techniques complemented with network analysis would cover the full range of expressed genes allowing to detect genes expressed at low levels and/or splice variants. This would provide unbiased information for identification of mechanistic bases of carotid plaque destabilization.

## Supporting Information

S1 Table **List of genes included in the study.** Probe gene sets with corresponding NM_number and probe ID (QIAGEN Ltd). FC, fold change (+, upregulated S vs A; -, downregulated S vs A).
(DOC)
