# Supplementary material for: Autophagic Marker MAP1LC3B Expression Levels Are Associated with Carotid Atherosclerosis Symptomatology
Source: PLoS One. 2014 Dec 12;9(12):e115176. doi: 10.1371/journal.pone.0115176 (PMC4264866; doi:10.1371/journal.pone.0115176)
Supplement: S1 Table — List of genes included in the study. Probe gene sets with corresponding NM_number and probe ID (QIAGEN Ltd). FC, fold change (+, upregulated S vs A; -, downregulated S vs A). (DOC) [file pone.0115176.s001.doc]

**Table S1: List of genes included in the study.** Probe gene sets with corresponding NM_number and probe ID (QIAGEN Ltd).

FC, fold change (+, upregulated S vs A; -, downregulated S vs A).

| **Gene Symbol** | **Gene Name** | **FC** | **P−value** | **Probe ID** | **NM_Number** |
| --- | --- | --- | --- | --- | --- |
| ANKRD55 | ankyrin repeat domain 55 | **+1.01** | **0.34** | QT01155392 | NM_001039935 NM_024669 |
| CALR | calreticulin | **+1.22** | **0.73** | QT00089215 | NM_004343 |
| CD163 | CD163 molecule | **+1.81** | **0.044*** | QT00074641 | NM_004244 NM_203416 |
| COL3A1 | collagen, type III, alpha 1 | **+1.42** | **0.063** | QT00058233 | NM_000090 |
| DDIT3 | DNA-damage-inducible transcript 3 | **+1** | **0.49** | QT00082278 | NM_001195053 NM_001195054 NM_001195055 NM_001195056 NM_004083 |
| DNAJB11 | DnaJ (Hsp40) homolog, subfamily B, member 11 | **+1.31** | **0.17** | QT00042560 | NM_016306 |
| DNAJB9 | DnaJ (Hsp40) homolog, subfamily B, member 9 | **−1.38** | **0.67** | QT00002716 | NM_012328 |
| DNAJC1 | DnaJ (Hsp40) homolog, subfamily C, member 1 | **−1.14** | **0.29** | QT00070182 | NM_022365 |
| DNAJC10 | DnaJ (Hsp40) homolog, subfamily C, member 10 | **−1.04** | **0.45** | QT00088529 | NM_018981 |
| DNAJC14 | DnaJ (Hsp40) homolog, subfamily C, member 14 | **−1.23** | **0.14** | QT00197043 | NM_032364 |
| EDN1 | endothelin 1 | **−1.07** | **0.44** | QT00088235 | NM_001955 |
| ELANE | elastase, neutrophil expressed | **−1.2** | **0.67** | QT00017010 | NM_001972 |
| ELN | elastin | **−1.01** | **0.23** | QT00034594 | NM_000501 NM_001081754 |
| ERO1LB | ERO1-like beta (S.cerevisiae) | **−1.67** | **0.034*** | QT00050456 | NM_019891 |
| ERP27 | endoplasmic reticulum protein 27 | **−4.07** | **0.47** | QT00025515 | NM_152321 |
| ERP29 | endoplasmic reticulum protein 29 | **+1.68** | **0.31** | QT00013153 | NM_006817 |
| ERP44 | endoplasmic reticulum protein 44 | **−1.05** | **0.42** | QT00025263 | NM_015051 |
| EVA1A | eva-1 homolog (C. Elegans) | **+1.57** | **0.033*** | QT00102879 | NM_032181 |
| FBXO4 | F-box protein 4 | **−1.05** | **0.29** | QT00033824 | NM_012176 |
| FKBP10 | FK506 binding protein 10, 65 kDa | **−1.11** | **0.1** | QT00075229 | NM_021939 |
| HERPUD1 | homocysteine-inducible, endoplasmic reticulum stress-inducible, ubiquitin-like domain member 1 | **−1.03** | **0.34** | QT00026418 | NM_001010989 NM_001010990 NM_014685 |
| HMGB1 | high mobility group box 1 | **−1.3** | **0.029*** | QT01002190 | NM_002128 |
| HMOX1 | heme oxygenase (decycling) 1 | **+2.07** | **0.065** | QT00092645 | NM_002133 |
| HSP90AA1 | heat shock protein 90kDa alpha (cytosolic), class A member 1 | **-1.29** | **0.12** | QT01848273 | NM_001017963 NM_005348 |
| HSP90B1 | heat shock protein 90kDa beta (Grp94), member 1 | **+1.17** | **0.07** | QT00046963 | NM_003299 |
| HSPA13 | heat shock protein 70kDa family, member 13 | **−1.14** | **0.047*** | QT00045325 | NM_006948 |
| HSPA1A | Heat shock 70kDa protein 1A | **−1.46** | **0.024*** | QT01002568 | NM_005345 |
| HSPA5 | heat shock 70kDa protein 5 (glucose-regulated protein, 78kDa) | **+1.03** | **0.23** | QT00096404 | NM_005347 |
| HYOU1 | hypoxia up-regulated 1 | **+1.37** | **0.1** | QT00046214 | NM_001130991 NM_006389 |
| IL10 | interleukin 10 | **−1.04** | **0.3** | QT00041685 | NM_000572 |
| IL12A | interleukin 12A (natural killer cell stimulatory factor 1, cytotoxic lymphocyte maturation factor 1, p35) | **−1.28** | **0.32** | QT00000364 | NM_000882 |
| IL12B | interleukin 12B (natural killer cell stimulatory factor 2, cytotoxic lymphocyte maturation factor 2, p40) | **−2.50** | **0.028*** | QT00000364 | NM_002187 |
| IL17A | interleukin 17A | **+1.07** | **0.15** | QT00009233 | NM_002190 |
| IL18 | interleukin 18 (interferon-gamma-inducing factor) | **+1.08** | **0.48** | QT00014560 | NM_001243211 NM_001562 |
| IL1A | interleukin 1, alpha | **+1.11** | **0.15** | QT00001127 | NM_000575 |
| IL23A | interleukin 23, alpha subunit p19 | **−1.18** | **0.09** | QT00204078 | NM_016584 |
| IL6 | interleukin 6 (interferon, beta 2) | **+1.24** | **0.2** | QT00083720 | NM_000600 |
| ITPR1 | inositol 1,4,5-triphosphate, type 1 | **+2.83** | **0.037*** | QT00056490 | NM_001099952 NM_001168272 NM_002222 |
| LMAN1 | lectin, mannose-binding, 1 | **−2.00** | **0.04*** | QT00065583 | NM_005570 |
| MANF | mesencephalic astrocyte-derived neurotrophic factor | **+1.68** | **0.48** | QT00050659 | NM_006010 |
| MAP1LC3B | microtubule-associated protein 1 light chain 3 beta | **−5.00** | **<0.0001**** | QT00055069 | NM_022818 |
| MAPK1 | mitogen-activated protein kinase 1 | **+1.75** | **0.057** | QT00065933 | NM_002745 NM_138957 |
| MMP9 | matrix metallopeptidase 9 (gelatinase B, 92kDa gelatinase, 92kDa type IV collagenase) | **+1.4** | **0.05*** | QT00040040 | NM_004994 |
| PARK2 | parkinson protein 2, E3 ubiquitin protein ligase (parkin) | **+1.52** | **0.043*** | QT00023401 | NM_004562 NM_013987 |
| PDIA3 | protein disulfide isomerase family A, member 3 | **−1.20** | **0.47** | QT00048776 | NM_005313 |
| PDIA4 | protein disulfide isomerase family A, member 4 | **+1.52** | **0.062** | QT00015883 | NM_004911 |
| PDIA6 | protein disulfide isomerase family A, member 6 | **+1.47** | **0.093** | QT00037086 | NM_005742 |
| RAB24 | RAB24, member RAS oncogene family | **−1.75** | **0.031*** | QT00200844 | NM_001031677 NM_130781 |
| RAF1 | v-raf-1 murine leukemia viral oncogene homolog 1 | **−1.67** | **0.074** | QT00038969 | NM_002880 |
| SEC63 | SEC63 homolog (S. cerevisiae) | **−1.46** | **0.05*** | QT00089719 | NM_007214 |
| SERP1 | stress-associated endoplasmic reticulum protein 1 | **−1.40** | **0.32** | QT00089327 | NM_014445 |
| SERPINH1 | serpin peptidase inhibitor, clade H (heat shock protein 47), member 1, (collagen binding protein 1) | **+1.06** | **0.42** | QT00044709 | NM_001207014 NM_001235 |
| SIL1 | SIL1 homolog, endoplasmic reticulum chaperone (S. cerevisiae) | **−1.27** | **0.01*** | QT00073262 | NM_001037633 NM_022464 |
| TGFB1 | transforming growth factor, beta 1 | **+1.57** | **0.13** | QT00000728 | NM_000660 |
| THBS1 | thrombospondin 1 | **+1.12** | **0.45** | QT00028497 | NM_003246 |
| TIMP1 | TIMP metallopeptidase inhibitor 1 | **+3.45** | **0.32** | QT00084168 | NM_003254 |
| TNF | tumor necrosis factor | **+1.11** | **0.13** | QT00029162 | NM_000594 |
| TXNDC12 | thioredoxin domein containing 12 (endoplasmic reticulum) | **−1.06** | **0.25** | QT00079177 | NM_015913 |
| VCAM1 | vascular cell adhesion molecule 1 | **+1.23** | **0.08** | QT00018347 | NM_001078 NM_001199834 NM_080682 |
